# The Response Characteristics of One *Saccharomyces cerevisiae* Strain Under Continuous Passage in Artificial Culture Medium

**DOI:** 10.3390/jof11070513

**Published:** 2025-07-09

**Authors:** Tengyu Ma, Hongguang Zhu, Jiajia Yin, Yu Tian, Wenjing Yan, Haixin Sun

**Affiliations:** College of Life Sciences, Qingdao University, Qingdao 266071, China; 19506109545@163.com (T.M.); hongg_zhu@163.com (H.Z.);

**Keywords:** *Saccharomyces cerevisiae*, isolation and screening, continuous passaging, biological characteristics, transcriptomics

## Abstract

*Saccharomyces cerevisiae* often undergoes strain degeneration during industrial serial subculturing, though this phenomenon remains understudied. This study first conducted strain screening and biological characterization through TTC (2,3,5-triphenyltetrazolium chloride) colorimetric assays, Durham tube fermentation gas production tests, and WL medium (Wallerstein Laboratory medium) cultivation. Subsequently, the changes in intergenerational biological traits after serial subculturing were investigated. Finally, transcriptomic analysis was employed to examine differential gene expression under high-glucose stress during continuous subculturing. The experimental results demonstrated that: (1) The *S. cerevisiae* QDSK310-Z-07 (GenBank: PP663884), isolated from farm soil, exhibited robust growth within a temperature range of 24–36 °C, with optimal growth observed at 28 °C. It thrived in a pH range of 4–5.5 and efficiently utilized various carbon and nitrogen sources; (2) After serial subculturing, the strain’s ethanol production capacity and fermentation rate partially declined and then stabilized, while maintaining strong tolerance to high ethanol concentrations and hyperosmotic stress; (3) Transcriptomic analysis revealed significant differential expression of genes related to lipid metabolism, amino acid metabolism, and other pathways under high-glucose stress following continuous subculturing. These findings elucidate the biological trait variations in *S. cerevisiae* during serial subculturing and provide key metabolic regulation candidate targets for its long-term adaptive evolution under high-glucose stress.

## 1. Introduction

*Saccharomyces cerevisiae* (*S. cerevisiae*), commonly known as brewer’s yeast, is a single-cell fungi. In 1996, Goffeau et al. completed its whole-genome sequencing, making it the first eukaryote to be fully sequenced [1]. Due to its clear genetic background, convenient molecular manipulation capabilities, and natural resistance to bacteriophage infections [2,3], *S. cerevisiae* is considered one of the most promising model chassis cells for industrial applications and fundamental research, widely used in various industries such as chemical engineering [4,5,6,7], food [8,9,10,11], and medical [12,13,14].

Excellent production strains must exhibit high ethanol yield, rapid fermentation, and robust stress tolerance to maximize efficiency and product quality [15,16,17,18]. During strain production and application, continuous subculturing is an inevitable process. Studies have demonstrated that serial passaging not only affects the genetic stability of strains but may also exert profound impacts on their metabolic characteristics [19]. During utilization, strains may exhibit degeneration phenomena, including alterations in original morphology, decreased nutrient utilization capacity, slowed growth rate, and weakened stress resistance [20,21,22]. These changes can subsequently manifest as consistently low yields during production and poor inter-batch reproducibility. Therefore, in-depth investigation of strain genetic stability holds significant theoretical and practical importance for maintaining the superior traits of original strains and ensuring stable, efficient industrial applications [23].

Current research on genetic stability during subculturing primarily focuses on probiotic strains. For instance, Wang et al. evaluated the genetic stability of *Lactiplantibacillus plantarum* P-8 through 100 generations of serial subculturing in MRS medium at 37 °C. Through morphological observation, carbohydrate utilization tests, and whole-genome sequencing analysis, they found no significant changes in cellular morphology or carbohydrate metabolism during passaging. Different generations maintained excellent genome collinearity with high similarity, demonstrating favorable genetic stability of *L. plantarum* P-8 during subculturing and establishing a genetic foundation for its industrial application (2023) [24]. Similarly, Liu et al. comprehensively assessed the genetic stability of *Bifidobacterium animalis* subsp. *lactis* V9 through phenotypic and genomic analyses. Their results showed no significant differences in cellular morphology or carbohydrate utilization across generations. Phylogenetic tree analysis revealed close genetic relationships between different passages, with no stably inherited mutation sites detected. These findings confirm that V9 maintains stable phenotypic and molecular genetic characteristics through 100 generations of continuous subculturing, providing crucial support for its further development and industrial-scale production (2023) [25].

However, current research on the genetic stability of yeast during serial subculturing remains relatively limited, highlighting the critical need for further investigation in this field. Strains of brewing yeast were isolated from farm soil, and after continuous subculturing, their biological characteristics were systematically characterized. Additionally, the transgenerational transcriptomic differences under high-sugar stress were elucidated, providing theoretical support for strain development and application. Compared to existing studies focusing on environmental stress responses in wild-type strains, these findings hold significant value for constructing microbial resource banks and exploring industrial potential.

## 2. Materials and Methods

### 2.1. Materials

The soil samples were collected from farm soils in Rizhao City, Shandong Province, China. YPD liquid medium, yeast agar, and WL medium were bought from Qingdao Hope Bio-Technology Co., Ltd., Qingdao, China. TTC medium was composed of upper and lower media layers. TTC upper medium comprised 5 g/L glucose (Sinopharm Chemical Reagent Co., Ltd., Shanghai, China), 0.5 g/L TTC (Shanghai Macleane Biochemical Technology Co., Ltd., Shanghai, China), and 15 g/L agar powder (Biosharp Biotechnology Co., Ltd., Anhui, China). TTC lower medium was composed of 10 g/L glucose, 1.5 g/L yeast extract (Sinopharm Chemical Reagent Co., Ltd., Shanghai, China), 2 g/L peptone (Beijing Oboxing Biotechnology Co., Ltd., Beijing, China), 0.4 g/L magnesium sulfate (Tianjin Beilian Fine Chemicals Development Co., Ltd., Tianjin, China), 1.0 g/L potassium dihydrogen phosphate (Sinopharm Chemical Reagent Co., Ltd.), and 20 g/L agar powder (pH 5.4–5.6). Anhydrous ethanol, galactose, lactose, soluble starch, citric acid, ethylamine hydrochloride, arginine, and ammonium chloride were all purchased from Sinopharm Chemical Reagent Co., Ltd. Glutathione was purchased from Shanghai Macleane Biochemical Technology Co., Ltd. Mannitol was purchased from Shanghai Aladdin Biochemical Technology Co., Ltd., Shanghai, China.

### 2.2. Methods

**Figure 1:**
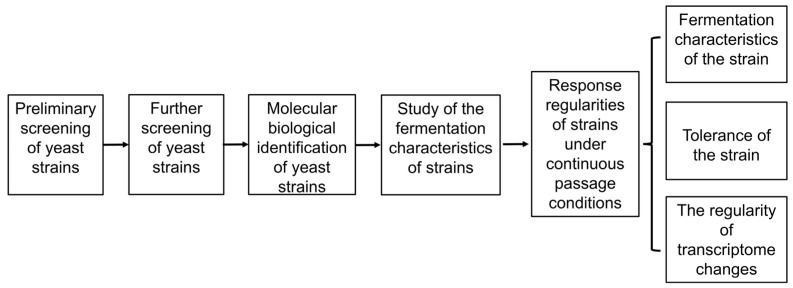
Experimental flow chart.

The experimental flow of this research is shown in Figure 1.

#### 2.2.1. Selection and Identification of Strains

(1) Preliminary screening of yeast strains

The initial bacterial suspension was serially diluted with sterile water at a 10-fold gradient. The diluted samples were spread onto yeast agar and incubated in a 28 °C electrically heated incubator (DHP-9052, Qingdao Lantenn Science and Education Instrument and Equipment Co., Ltd., Qingdao, China) for 24–48 h. Isolation and purification were carried out following established protocols, and the resulting strains were numbered and preserved [26,27,28].

2,3,5-triphenyltetrazolium chloride (TTC) chromogenic experiments: Isolated yeast strains were streaked onto the lower layer of TTC medium and incubated at 28 °C for 3 days. After colony formation, the upper TTC medium was poured over the colonies and incubated at 28 °C in the dark for 3 h [29]. Strains with darker colony colors were selected for the gas production fermentation experiment [28].

Experiments on gas production by Durham tubule fermentation: By monitoring the gas-filling state of the Durham tube and the time required for complete gas accumulation, the fermentation rate and gas-producing capability of the strain can be evaluated [28,30]. An amount of 10 mL YPD liquid medium was aliquoted into 15 mL test tubes, inverted, and placed in Durham tubes. After sterilization (vertical autoclave, YXQ-LS-50SI, Shanghai Boxun Industrial Co., Ltd. Medical Equipment Factory, Shanghai, China), and cooling, a 5% inoculum was added, and the tubes were incubated at 28 °C. The gas volume and time required to fill the Durham tubes were recorded every 6 h [30]. Based on the preliminary screening results, strains with strong alcohol production, rapid fermentation, and high gas production were selected for further screening.

(2) Further screening of yeast strains

The selected strains were streaked onto WL medium and incubated at 28 °C for 3–5 days. After colony formation, the strains were preliminarily identified based on the colony morphology observed on the WL medium [31].

(3) Molecular biological identification of yeast strains

Single colonies isolated from culture media were subjected to DNA extraction. After successful extraction, PCR amplification and agarose gel electrophoresis were performed, and the product was sequenced. The sequencing results were compared with homologous sequences using the Basic Local Alignment Search Tool (BLAST) from the National Center for Biotechnology Information (NCBI). Finally, the phylogenetic tree of the strains was constructed using MEGA7.0.26.

#### 2.2.2. Study of the Fermentation Characteristics of Strains

Yeast strains were cultured in YPD liquid medium (28 °C, 150 rpm) to measure OD_600_ values (ultraviolet spectrophotometer, UV-8000, Shanghai Yuanxi Instrument Co., Ltd., Shanghai, China) and generate growth curves. The ability of yeast strains to utilize various carbon sources (glucose, galactose, lactose, soluble starch, citric acid, and mannitol) and nitrogen sources (ethylamine hydrochloride, arginine, peptone, glutathione, and ammonium chloride) was evaluated by Durham tubes fermentation test. Strain adaptability was assessed across temperature gradients (20–40 °C) and pH ranges (3.5–5.5) [32,33].

#### 2.2.3. Response Regularities of Strains Under Continuous Passage Conditions

The selected and identified strains were continuously passed up to 30 generations, designated G1, G2, and G30, respectively. The biological characteristics of G1, G5, G10, G15, G20, G25, and G30 were compared.

(1) Fermentation characteristics of the strain.

Durham tubule fermentation gas production experiment and TTC chromogenic experiment were used to explore the changes in fermentation ability of strains after passage.

(2) Tolerance of the strain.

Anhydrous ethanol was added to the YPD liquid medium at different concentrations (4%, 6%, 8%, 10%, and 12%). The medium was inoculated with 1% inoculum and incubated at 28 °C for 48 h, and the OD_600_ of the fermentation liquid was measured [17,34,35].

Based on the yeast strain’s utilization of different carbon and nitrogen sources, solutions of the most efficiently utilized carbon and nitrogen sources were prepared at 200 g/L, 250 g/L, 300 g/L, 350 g/L, and 400 g/L [36]. The experimental parameters were the same as those in the carbon and nitrogen source assimilation capacity study, and the OD_600_ of the fermentation liquid was measured.

(3) The regularity of transcriptome changes.

In view of the differences in the growth conditions of different generations of *S. cerevisiae* QDSK310-Z-07 at a glucose concentration of 400 g/L, transcriptome analysis of G1, G15, and G30 was carried out in this research. After obtaining all raw sequences from the second-generation, high-throughput sequencing platform, the sequences were processed to remove low-quality reads and adapter contamination, resulting in high-quality sequences. After the transcriptome data quality assessment was qualified, comprehensive analysis was performed using Gene Ontology (GO) and the Kyoto Encyclopedia of Genes and Genomes (KEGG) databases [37,38,39].

### 2.3. Data Analysis

All experiments were performed in triplicate replicates, the experimental data were analyzed and processed by SPSS 27.0 software, the one-way ANOVA was analyzed using Duncan’s test, and Origin2021 and MEGA 7.0.26 software generated the graphs.

## 3. Results

### 3.1. Screening and Identification of Strains

Following enrichment culture, isolation, and purification, 14 suspected yeast strains were obtained from farm soils samples. These strains were designated as Y1, Y2, Y3, Y4, Y5, Y6, Y7, Y8, Y9, Y10, Y11, Y12, Y13, and Y14.

The 14 strains obtained after isolation and purification were subjected to TTC colorimetric experiments. The results are shown in Table 1. Strains Y1, Y3, Y4, Y5, and Y7, which exhibited strong alcohol production capacity, were selected for the fermentation gas production experiment.

The results of the gas production test are shown in Table 2. At 12 h, strains Y5 and Y7 exhibited the highest gas accumulation in Durham tubes, followed by strains Y3 and Y4, while strain Y1 showed the least gas production. By 24 h, the Durham tubes for strains Y3, Y4, Y5, and Y7 had reached full gas saturation, whereas the Durham tube of strain Y1 remained incompletely filled. These findings indicate that strains Y3, Y4, Y5, and Y7 exhibit both superior fermentation capacity and faster gas production rates. According to the preliminary screening results, strains Y3, Y4, Y5, and Y7 with strong alcohol production and fermentation ability were selected for further screening.

**Figure 2:**
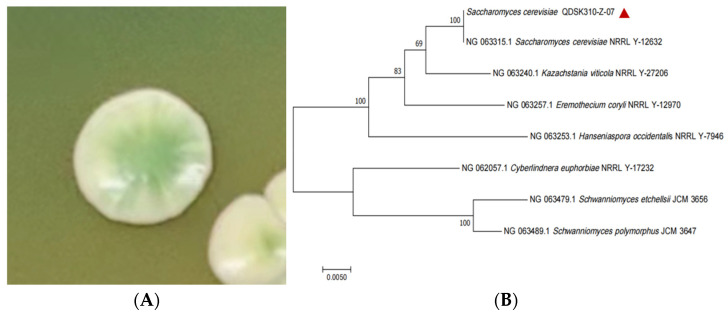
(**A**) Colony map of strain Y7 on WL medium. (**B**) Phylogenetic tree of *S. cerevisiae* QDSK310-Z-07 based on the 18S rRNA gene sequence.

As presented in Table 3, the morphological characteristics of the colonies of strains Y3, Y5, and Y7 on WL medium were similar to those of *S. cerevisiae* on WL medium in the literature, whereas strain Y4 displayed distinct morphological features [31]. Therefore, based on the results of both preliminary and re-screening tests, strain Y7, which has strong ethanol production and fermentation abilities, was chosen for molecular identification. The colonies of strain Y7 cultured on WL medium are shown in Figure 2A.

Phylogenetic tree (Figure 2B) revealed that strain Y7 clustered with *S. cerevisiae* NRRL Y-12632, indicating high homology. Strain Y7 was ultimately identified as *S.cerevisiae*, registered with GenBank under accession number PP663884, and renamed *S. cerevisiae* QDSK310-Z-07.

### 3.2. Fermentation Characteristics of S. cerevisiae QDSK310-Z-07

**Figure 3:**
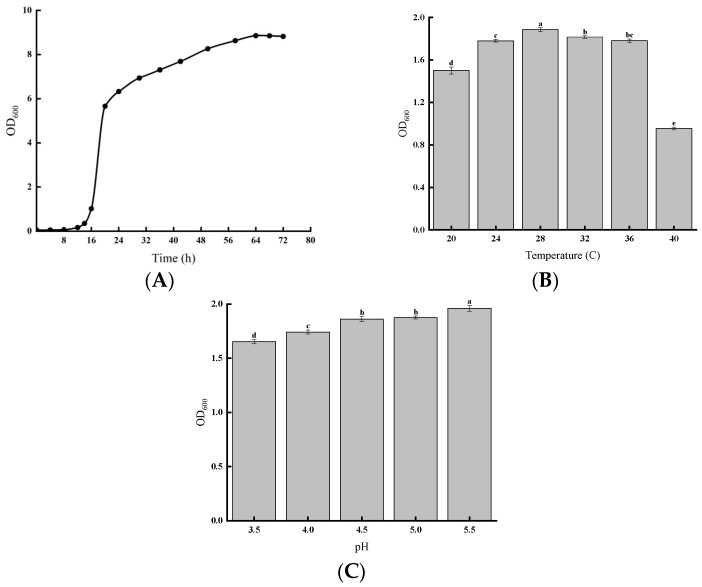
(**A**) Growth curve of *S. cerevisiae* QDSK310-Z-07. (**B**) Growth of *S. cerevisiae* QDSK310-Z-07 at different temperatures. (**C**) Growth of *S. cerevisiae* QDSK310-Z-07 at different pH levels.

As illustrated in Figure 3A, the yeast strain was in the lag phase from 0 to 14 h, with slow growth. From 14 to 24 h, it entered the logarithmic growth phase, followed by the stationary phase from 24 to 64 h. After 64 h, the strain gradually entered the death phase. Moreover, *S. cerevisiae* QDSK310-Z-07 exhibited good growth between 24 °C and 36 °C, with optimal growth at 28 °C (Figure 3B). Growth was strongly inhibited at 40 °C. As the pH value decreased, making the environment more acidic, the growth of *S. cerevisiae* QDSK310-Z-07 was increasingly inhibited (Figure 3C).

Through Table 4, we can visually compare the gas production of *S. cerevisiae* QDSK310-Z-07 under different carbon and nitrogen sources, thereby elucidating the strain substrate utilization capacities. For example, when glucose is used as the only carbon source, there is gas in the Durham tube at 12 h, and the gas filling state is reached at 24 h, while when citric acid is used as the only carbon source, there is no gas in the Durham tube at 12 h, and there is only a small amount of gas at 24 h. Therefore, from Table 4, we can conclude that *S. cerevisiae* QDSK310-Z-07 has poor utilization of soluble starch and citric acid, while its assimilation of galactose and glucose is more significant compared to other carbon sources. Among nitrogen sources, yeast has the strongest degree of assimilation of peptone.

### 3.3. Response Regularities of S. cerevisiae QDSK310-Z-07 Under Continuous Passage Conditions

The changes in the fermentation ability and gas production rate of *S. cerevisiae* QDSK310-Z-07 after continuous passage were explored by Durham tube gas production experiment, and the greater the gas accumulation in the Durham tube within equivalent time intervals, the stronger the fermentation ability of the strain. After G5, the fermentation power of the strain slightly increased in G10 and G15, then gradually decreased (Table 5). The changes in alcohol production capacity of *S. cerevisiae* after continuous passage were explored by the degree of color development on the colony on TTC medium, and the darker the color rendering, the stronger the alcohol production ability. After G10, the ethanol production capacity of the strain slightly increased in G15 and then stabilized at a moderate level (Table 6).

**Figure 4:**
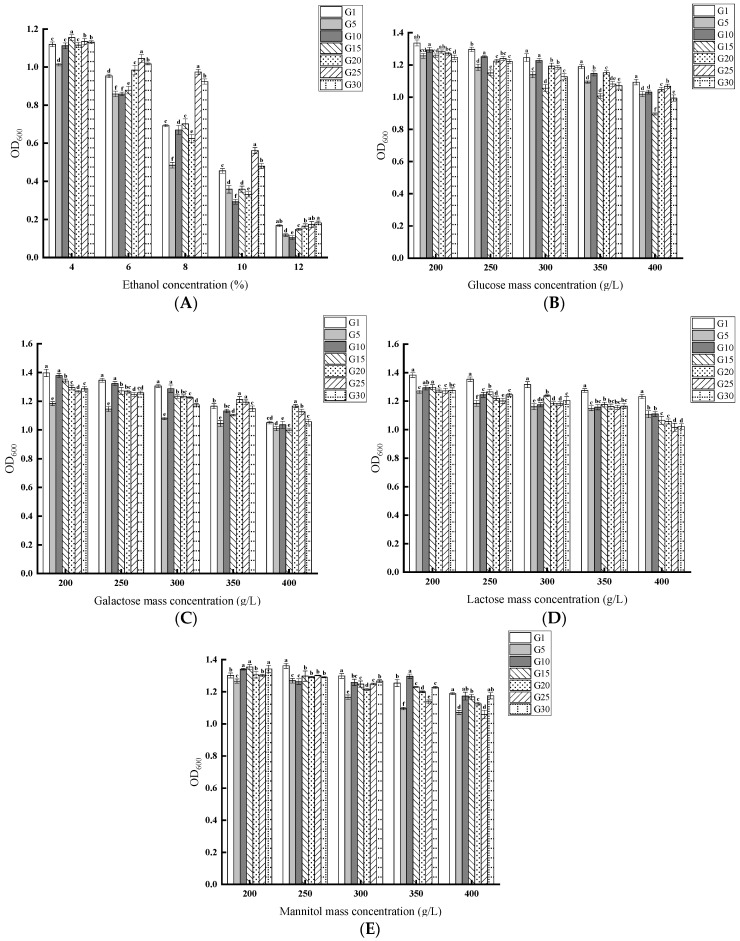
(**A**): Ethanol tolerance of *S. cerevisiae* QDSK310-Z-07 across different generations. Effect of different concentrations of carbon sources on different generations of *S. cerevisiae* QDSK310-Z-07. (**B**) Glucose; (**C**) Galactose; (**D**) Lactose; (**E**) Mannitol.

As presented in Figure 4A, with increasing ethanol concentration, the growth of the strain was gradually inhibited. After successive passages, the strain growth was progressively suppressed, but the degree of inhibition varied across generations. For instance, G5 was more strongly inhibited at the same ethanol concentration than the others.

With increasing glucose concentration, the growth of the strain was progressively inhibited. After successive passages, the strain’s growth was progressively suppressed, but the degree of inhibition varied across generations. At the same concentration, G15 was more strongly inhibited by high glucose levels than others (Figure 4B). With increasing galactose concentration, the growth of the strain was progressively inhibited. At the same concentration, G5 was more strongly inhibited than others (Figure 4C). As lactose concentration increased, the growth of the yeast was progressively inhibited, and the degree of inhibition varied across different generations under the same concentration conditions (Figure 4D). As mannitol concentration increased, the growth of the yeast was progressively inhibited. Under the same concentration, G5 was more strongly inhibited, similarly to the phenomenon observed when galactose was the sole carbon source (Figure 4E).

**Figure 5:**
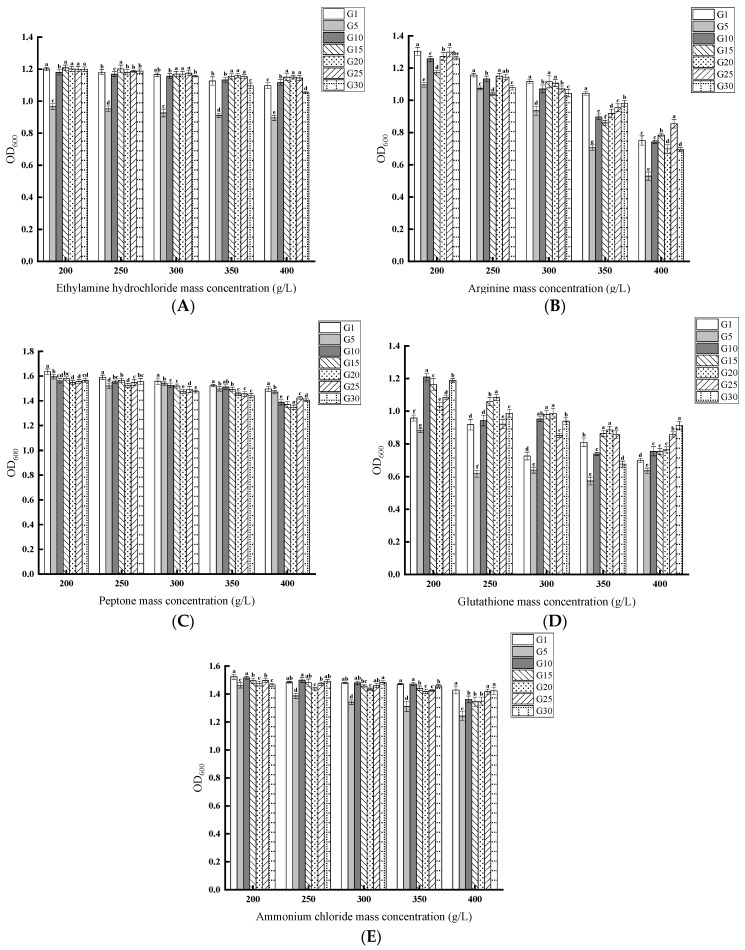
Effect of different concentrations of nitrogen sources on different generations of *S. cerevisiae* QDSK310-Z-07. (**A**) Ethylamine hydrochloride; (**B**) Arginine; (**C**) Peptone; (**D**) Glutathione; (**E**) Ammonium chloride.

Yeast growth was not strongly inhibited at an ethylamine hydrochloride concentration of 200 g/L to 400 g/L. However, at the same concentration, G5 was more strongly inhibited than others (Figure 5A). As arginine concentration increased, the growth of the yeast was progressively inhibited. Under the same concentration, G5 was more strongly inhibited than others (Figure 5B). Yeast growth was not strongly affected at a 200 g/L to 400 g/L peptone concentration. Comparing OD_600_ across different nitrogen sources at the same concentration gradient, yeast growth was better when peptone was used as the nitrogen source (Figure 5C). As glutathione concentration increased, the growth of the yeast was progressively inhibited. Relative to other generations, G5 was more strongly inhibited under the same concentration conditions (Figure 5D). Within the range of ammonium chloride concentrations from 200 g/L to 400 g/L, yeast growth was not significantly inhibited. However, G5 experienced more pronounced inhibition under the same concentration conditions than other generations (Figure 5E).

### 3.4. Results of Transcriptomic Studies of S. cerevisiae

**Figure 6:**
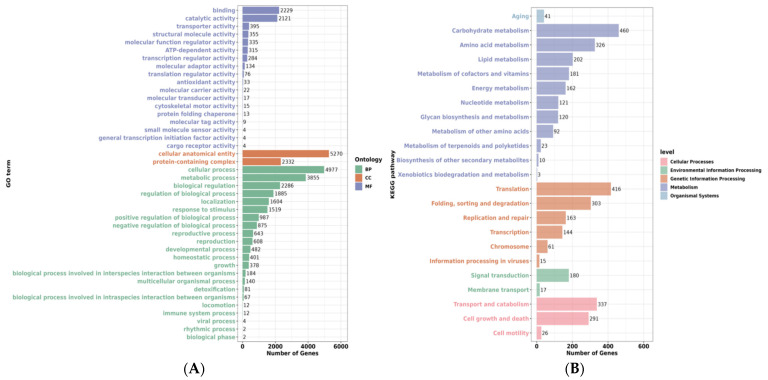
(**A**) GO annotation result graph of *S. cerevisiae* QDSK310-Z-07 under high glucose stress. (**B**) KEGG annotation result graph of *S. cerevisiae* QDSK310-Z-07 under high glucose stress.

The GO functional annotation analysis revealed a multidimensional stress response mechanism of *S. cerevisiae* QDSK310-Z-07 under high-glucose stress (Figure 6A). At the molecular function (MF) level, genes associated with binding and catalytic activity predominated, indicating that cells cope with high-sugar stress by enhancing metabolic enzyme activity and molecular interactions. In the cellular component (CC) category, genes related to cellular anatomical entities and protein complexes were predominant, reflecting an active process of dynamic adjustment in organelle structures. Regarding biological processes (BP), the high frequency of genes involved in metabolic processes, cellular processes, and biological regulation unveiled a systemic cellular response in energy conversion and homeostasis regulation. Notably, the presence of stress-related terms, such as genes linked to detoxification and antioxidant activity, may suggest an adaptive mechanism in yeast cells induced by the high-glucose environment. As illustrated in Figure 6B, the KEGG annotation map reveals a multidimensional stress response mechanism in *S. cerevisiae* QDSK310-Z-07 cells under high-glucose stress. Metabolic Network Remodeling: Genes associated with carbohydrate metabolism, amino acid metabolism, and lipid metabolism pathways were frequently annotated, suggesting that cells may undergo metabolic flux redistribution to adapt to the high-sugar environment. Activation of Stress Defense Systems: The annotation of genes involved in energy metabolism, cofactors and vitamins metabolism pathways reflects an enhanced oxidative stress defense and coenzyme regeneration mechanism under high-glucose conditions. Maintenance of Genetic Information: The significant annotation of genes related to transcription, translation, protein folding and degradation, as well as DNA replication and repair pathways, indicates that cells may reinforce protein quality control to maintain functional homeostasis under high-glucose stress. Signal Regulation Network: The frequent annotation of genes associated with signal transduction and membrane transport pathways suggests an adaptive regulatory mechanism for sensing and transmitting high-glucose signals.

**Figure 7:**
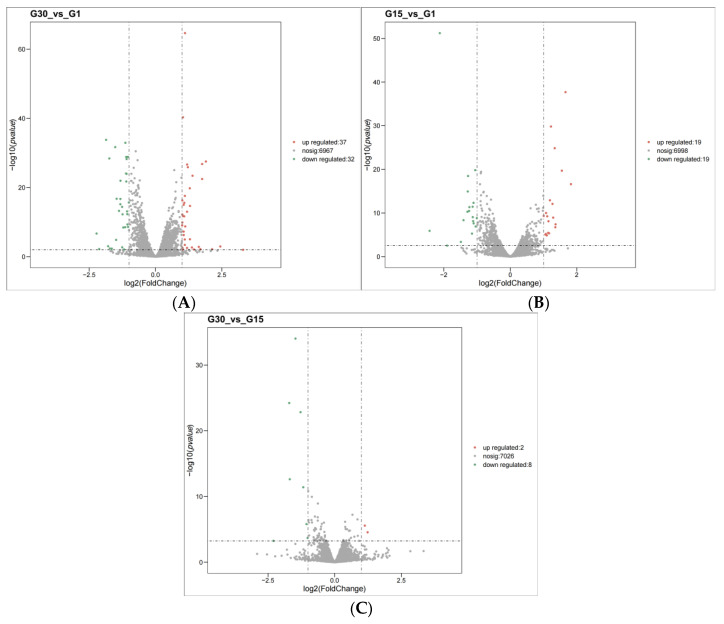
Volcano plot of differential expression genes under high glucose stress. (**A**) G30 vs. G1; (**B**) G15 vs. G1; (**C**) G30 vs. G15.

In comparison with G30, G1 had 37 upregulated genes and 32 downregulated genes (Figure 7A). Compared to G15, G1 had 19 upregulated and 19 downregulated genes (Figure 7B). Relative to G30, G15 had 2 upregulated and 8 downregulated genes (Figure 7C). Figure 5 contains a substantial number of genes without significant differential expression, indicating the stability of core metabolic pathways during serial passaging. The significantly differentially expressed genes require further inference through GO and KEGG enrichment analyses.

**Figure 8:**
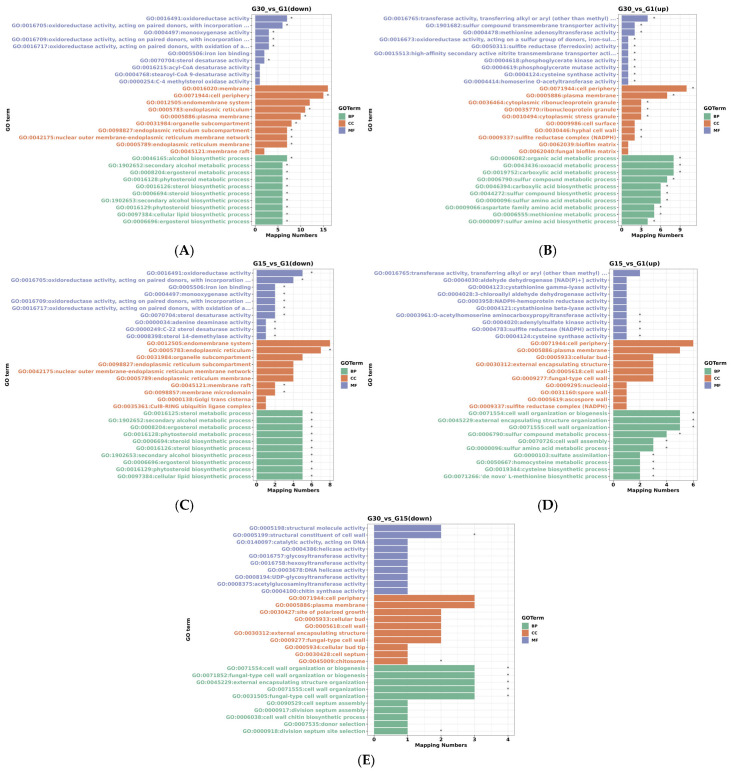
Bar chart of GO enrichment analysis for differential expression genes under high glucose stress. (**A**,**B**) G30 vs. G1; (**C**,**D**) G15 vs. G1; (**E**) G30 vs. G15. * indicates significant expression of genes corresponding to this pathway.

Relative to G30, the differential expression genes (DEGs) in G1 were significantly enriched in processes such as lipid biosynthesis and metabolism, amino acid biosynthesis and metabolism, organic acid metabolism, and secondary alcohol metabolism. In terms of cellular components, the DEGs showed significant enrichment in subcategories including the cell periphery, endoplasmic reticulum, and cytoplasmic stress granules. Regarding molecular functions, they were notably enriched in enzymatic activity (Figure 8A,B). In contrast to G15, the DEGs in G1 were significantly enriched in lipid biosynthesis and metabolism, amino acid biosynthesis and metabolism, cell wall organization or biogenesis, and secondary alcohol metabolism. For cellular components, the DEGs exhibited significant enrichment in membrane microdomains and the endoplasmic reticulum. As for molecular functions, they were markedly enriched in enzymatic activity (Figure 8C,D). When compared to G30, the DEGs in G15 were significantly enriched in processes such as cell wall organization or biogenesis. In cellular components, the DEGs were notably enriched in chitosomes, while in molecular functions, they were predominantly enriched in cell wall structural constituents (Figure 8E).

**Figure 9:**
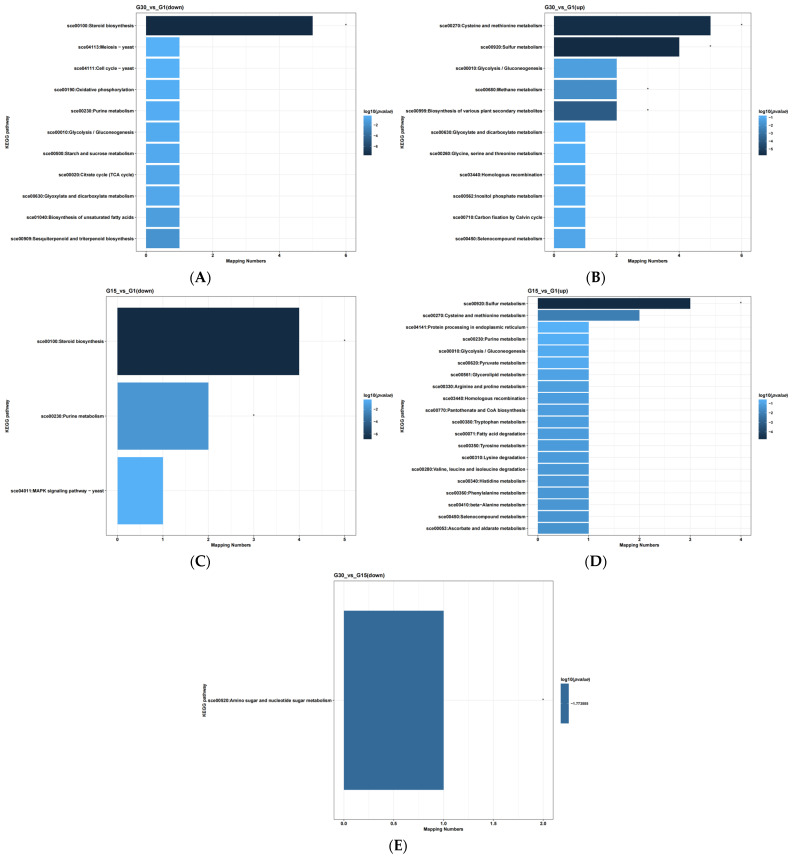
Bar chart of KEGG enrichment analysis for differential expression genes under high glucose stress. (**A**,**B**), G30 vs. G1; (**C**,**D**), G15 vs. G1; (**E**), G30 vs. G15. * indicates significant expression of genes corresponding to this pathway.

Relative to G30, the DEGs in G1 were significantly enriched in pathways including steroid biosynthesis, cysteine and methionine metabolism, sulfur metabolism, methane metabolism, and the biosynthesis of various secondary metabolites (Figure 9A,B). Unlike G15, the DEGs in G1 were significantly enriched in steroid biosynthesis, purine metabolism, and sulfur metabolism pathways (Figure 9C,D). In contrast to G30, the DEGs in G15 were significantly enriched in amino sugar and nucleotide sugar metabolism pathways (Figure 9E).

## 4. Discussion

Industrial *S. cerevisiae* used in production has many excellent characteristics, such as high ethanol production capacity, strong tolerance, and genetic stability [33,40,41,42,43]. Therefore, studying the characteristics of independently selected strains is particularly important. Carbon and nitrogen sources are essential substances for microbial life activities, and different carbon and nitrogen sources have varying effects on yeast. The ability of yeast to utilize different carbon and nitrogen sources also varies. Experiments showed that *S. cerevisiae* QDSK310-Z-07 has strong glucose, galactose, and peptone assimilation ability. In contrast, its ability to assimilate mannitol, ethylamine hydrochloride, glutathione, and ammonium chloride is moderate, and it has poor assimilation ability for soluble starch and citric acid. Aké et al. systematically investigated the assimilation and fermentation capabilities of multiple yeast strains toward six carbon sources: glucose, galactose, fructose, lactose, sucrose, and maltose. Unlike our study, all strains in the Aké et al. research could assimilate sucrose and glucose but could not assimilate lactose. Some strains, like *Pichia kudriavzevii* AR 2-32-2 and *Pichia manshuurica* RE 2-2, could not assimilate galactose (2019) [44]. Yeast prioritizes easily decomposed substances with lower ATP consumption when various carbon sources are present, supporting our findings from carbon source assimilation experiments [33,41].

Temperature affects the survival rate and fermentation rate of yeast, and the yeast strains used in fermentation should exhibit good thermal stability [45]. Our experimental results demonstrated that the *S. cerevisiae* QDSK310-Z-07 exhibited robust growth within the temperature range of 24–36 °C, with 28 °C identified as its optimal growth temperature. This thermal adaptation characteristic aligns with the findings of Zhang et al., whose studied yeast strain Y2 similarly showed optimal growth at 28 °C (2020) [28]. Notably, low pH conditions during fermentation were observed to significantly inhibit yeast growth and metabolic activity, ultimately leading to complete fermentation arrest. These findings underscore the importance of investigating pH adaptation characteristics for industrial applications of this strain. When the pH range was 3.5–5.5, and the yeast strain was cultured at 28 °C for 24 h, growth was gradually inhibited as the pH decreased. The trend of growth inhibition at different pH conditions was consistent with the results of Olee et al. (2022) [46]. In the laboratory, the TTC colorimetric method is commonly employed to assess the alcohol-producing capacity of yeast strains. The principle of this method lies in the reaction between the metabolic byproducts of yeast strains and the chromogenic agent TTC. A more intense coloration indicates higher respiratory enzyme activity in the yeast strain, which corresponds to greater alcohol production capability [29]. The colorimetric analysis on TTC medium revealed distinct ethanol production capacities among different generations of yeast strains. Strain G1 and G5 maintained high-yield ethanol production levels after continuous subculturing. Following G10, strain G15 exhibited a modest enhancement in ethanol production capacity, which subsequently stabilized at medium-yield levels. These findings demonstrate relatively stable ethanol-producing performance throughout the serial passaging process. Gas production experiments showed that fermentation capacity slightly increased in G10 and G15 after G5, but fermentation capacity gradually decreased with continuous subculturing. The specific reasons for this decline need further investigation at the metabolomic and transcriptomic levels.

Ethanol is a fermentation product of *S. cerevisiae*, but high ethanol concentrations can lead to protein denaturation, decreased cell viability, increased mortality, and subsequently affect the fermentation process [47,48]. The high ethanol tolerance of *S. cerevisiae* facilitates complete fermentation during the brewing process and increases the alcohol content of the product. We found that growth across different yeast generations gradually decreased as ethanol concentration increased. *S. cerevisiae* QDSK310-Z-07 showed strong growth in environments with ethanol concentrations of 4–8%, but growth significantly slowed at concentrations exceeding 10%. Therefore, the yeast strain exhibited ethanol tolerance up to 8%. This growth trend was consistent with the results of Long et al., and the ethanol tolerance of the strains screened in this research was better than that of the yeast strains screened by Long et al. (6% ethanol tolerance) (2024) [31]. Additionally, G5 and G10 showed more potent growth inhibition under the same ethanol concentration than other generations.

Current research on carbon and nitrogen sources for *S. cerevisiae* mainly focuses on utilizing different carbon and nitrogen sources, with limited exploration of yeast growth after continuous subculturing under varying concentrations of carbon and nitrogen sources. This research examined the growth of different yeast generations under different concentrations of carbon and nitrogen sources based on their utilization of these sources. Increased concentrations of carbon and nitrogen sources affected yeast growth to varying degrees, and the inhibitory effect on different yeast generations varied under the same carbon and nitrogen source concentration. For instance, generation G5 exhibited more significant inhibition under the same arginine concentration compared to other generations.

During fermentation, high glucose concentrations create a hyperosmotic environment, leading to yeast cell rupture, water loss, and reduced activity, inhibiting yeast growth, potentially altering metabolism, or making fermentation difficult [49,50,51]. As glucose concentration increased, the growth of different generations of *S. cerevisiae* QDSK310-Z-07 was increasingly inhibited. This trend is consistent with the findings of Long et al., where yeast strain LJM-4 growth was gradually inhibited as glucose concentration increased from 200 g/L to 350 g/L and then to 400 g/L (2024) [31]. However, under the same glucose concentration, the reasons for the different degrees of inhibition of different generations of strains need to be further explored.

Transcriptomics was used to study the gene expression differences of different generations of *S. cerevisiae* at a glucose concentration of 400 g/L. The proportion of genes in different functional categories reflects the metabolic or physiological biases under specific periods and conditions. The transcriptome results of G1, G15, and G30 showed that the expression of genes related to amino acid metabolism, amino sugar and nucleotide sugar metabolism, and lipid metabolism was significantly different in the continuous passage of *S. cerevisiae* QDSK310-Z-07 in a high glucose environment. Consistent with existing theories, our experimental results confirm that yeast cells counteract high-sugar stress through coordinated mechanisms, such as ergosterol metabolism, membrane permeability regulation, and signal transduction activation [38,52,53]. It was inferred that when the glucose concentration was 400 g/L, compared with G1 and G30, the growth of G15 was strongly inhibited, which was significantly related to the significant expression of differential genes. To further investigate the molecular mechanisms underlying this high-glucose tolerance, key DEGs (Table A1) identified from major metabolic pathways should be validated via quantitative real-time PCR (qPCR). This follow-up experiment would elucidate the relationship between the expression levels of these critical genes and the strain’s osmotolerant phenotype, providing deeper insights into its adaptive responses.

Based on the experimental results obtained in this research, future research could explore the following directions in greater depth:

(1) Regarding the environmental stress tolerance exhibited by *S. cerevisiae* QDSK310-Z-07, genetic engineering approaches could be employed to enhance the expression of stress-resistant genes. For instance, gene editing technologies could be utilized to precisely regulate the expression levels of stress-related genes, while adaptive laboratory evolution strategies could be integrated to develop superior strains with multi-stress tolerance. This would enable the strain to thrive in the complex conditions of industrial production, ultimately cultivating strains with higher industrial applicability.

(2) This study primarily examined the biological characteristics of *S. cerevisiae* QDSK310-Z-07 at G1, G5, G10, G15, G20, G25, and G30 generations. Building upon these findings, more detailed and in-depth investigations could be conducted to comprehensively assess the strain’s genetic stability and fermentation performance over prolonged serial passages. Such research would help evaluate its potential as a standard reference strain and lay a theoretical foundation for establishing a strain seed bank.

## 5. Conclusions

We isolated *S. cerevisiae* strains from farm soil and investigated their biological characteristics at both macroscopic and microscopic levels. The main findings are as follows:

(1) The farm soils were used as the isolation source, and the strain of *S. cerevisiae* QDSK310-Z-07 was independently screened. The strain exhibits broad adaptive growth characteristics: it grows well within a temperature range of 24–36 °C, with an optimal growth temperature of 28 °C. It maintains stable growth at pH 4.0–5.5 and demonstrates efficient utilization of various carbon and nitrogen sources. These findings offer novel microbial resources with potential industrial applications.

(2) The study investigated the changes in alcohol production capacity, fermentation rate, and tolerance to products and various substrates of the strain after subculturing. After continuous subculturing, the strain’s ethanol tolerance and fermentation capacity decreased, with ethanol production capacity gradually stabilizing at a medium level. With increase in concentration, the tolerance to various carbon and nitrogen sources and ethanol decreased to varying degrees. The results delineate the strain’s adaptive response patterns following serial passaging, offering novel research avenues for investigating genetic stability while establishing a robust foundation for developing strain seed banks and advancing yeast strain applications.

(3) Transcriptomics analysis revealed differences in gene expression related to lipid metabolism and amino acid metabolism in yeast strains sub-cultured under high-sugar environments. These findings provide a crucial theoretical foundation for the industrial application of this strain, while also offering clear guidance for subsequent strain improvement and fermentation process optimization.

## Figures and Tables

**Table 1:** Results of TTC chromogenic experiments.

Number	Colony Coloring	Number	Colony Coloring
Y1	Red	Y8	Light red
Y2	Light pink	Y9	Pink
Y3	Red	Y10	Light red
Y4	Deep red	Y11	Pink
Y5	Red	Y12	Light pink
Y6	Light pink	Y13	Pink
Y7	Deep red	Y14	Light pink

**Table 2:** Results of the gas production experiments.

Strain Number	12 h	24 h
Y1	+	+++
Y3	++	++++
Y4	++	++++
Y5	+++	++++
Y7	+++	++++

Note: +, ++, +++ indicate gas volumes occupying one-third, one-half, and four-fifths of the Durham tube, respectively. ++++ indicates the Durham tube is completely filled with gas.

**Table 3:** Colony characteristics of strains on WL medium.

Strain Number	Colony Characteristics
Y3	The colony edges are creamy white, the center is light green, and the surface is smooth.
Y4	The colony surface is rough, and the colony appears grayish-green.
Y5	The colony surface is smooth, with spherical protrusions. The center is light green and creamy.
Y7	The colony is relatively large, with creamy edges and a green center, and has a smooth surface.

**Table 4:** Gas production results for different carbon and nitrogen sources in *S. cerevisiae* QDSK310-Z-07.

Carbon Source	12 h	24 h	Nitrogen Source	12 h	24 h
Glucose		++++	Ethylamine hydrochloride	—	++++
Galactose	·	++++	Arginine	—	++++
Lactose	—	++++	Peptone	·	++++
Soluble starch	—	+	Glutathione	—	++++
Citric acid	—	+	Ammonium chloride	—	++++
Mannitol	—	++++			

Note: — indicates no gas production; · indicates a trace amount of gas; + indicates gas volume occupying one-fifth of the Durham tube; ++++ indicates the Durham tube is completely filled with gas.

**Table 5:** Gas production results of *S. cerevisiae* QDSK310-Z-07.

Strain Algebra	12 h	24 h
G1	+++	++++
G5	++	++++
G10	+··	++++
G15	+··	++++
G20	++	++++
G25	+	++++
G30	—	++++

Note: +, ++, +··, +++ indicate gas volumes occupying one-fifth, one-half, two-thirds, and four-fifths of the Durham tube, respectively; — indicates no gas production; ++++ indicates the Durham tube is completely filled with gas.

**Table 6:** Ethanol production results of *S. cerevisiae* QDSK310-Z-07.

Strain Algebra	Colony Coloring
G1	Deep Red
G5	Deep Red
G10	Red
G15	Deep Red
G20	Red
G25	Red
G30	Red

## Data Availability

The original contributions presented in this study are included in the article. Further inquiries can be directed to the corresponding author.

## Abbreviations

The following abbreviations are used in this manuscript:

WL medium	Wallerstein Laboratory medium
TTC	2,3,5-triphenyltetrazolium chloride
YPD liquid medium	Yeast extract peptone dextrose liquid medium
NCBI	National Center for Biotechnology Information
BLAST	Basic Local Alignment Search Tool
GO	Gene Ontology
KEGG	Kyoto Encyclopedia of Genes and Genomes
DEGs	Differential Expression Genes

## Appendix A

**Table A1:** *S. cerevisiae* QDSK310-Z-07 at a glucose concentration of 400 g/L for some of the key DEGs. (*padj* < 0.05).

Gene ID	Name	Description
YNL192W	*CHS1*	chitin synthase CHS1
YGR060W	*ERG25*	methylsterol monooxygenase
YHR007C	*ERG11*	sterol 14-demethylase
YLR056W	*ERG3*	C-5 sterol desaturase
YMR015C	*ERG5*	C-22 sterol desaturase
YIR027C	*DAL1*	allantoinase
YNL141W	*AAH1*	adenine deaminase
YFR030W	*MET10*	sulfite reductase subunit alpha
YGR175C	*ERG1*	squalene monooxygenase
YKL001C	*MET14*	adenylyl-sulfate kinase
YLR303W	*MET17*	bifunctional cysteine synthase/O-acetylhomoserine aminocarboxypropyltransferase MET17
YJR137C	*MET5*	sulfite reductase (NADPH) subunit beta
YNL277W	*MET2*	homoserine O-acetyltransferase
YDR502C	*SAM2*	methionine adenosyltransferase SAM2
YGL184C	*STR3*	cystathionine beta-lyase STR3
YLR180W	*SAM1*	methionine adenosyltransferase SAM1
YHL008C	*YHL008C*	uncharacterized protein
YKL152C	*GPM1*	phosphoglycerate mutase GPM1
YNL111C	*CYB5*	Cyb5p
YGL055W	*OLE1*	stearoyl-CoA 9-desaturase
YBR078W	*ECM33*	Ecm33p
YPR194C	*OPT2*	Opt2p
YPR192W	*AQY1*	Aqy1p
YPL036W	*PMA2*	H (+)-exporting P2-type ATPase PMA2
YPL265W	*DIP5*	dicarboxylic amino acid permease
YKL096W-A	*CWP2*	Cwp2p
YHR154W	*RTT107*	Rtt107p
YHR149C	*SKG6*	Skg6p
YGR279C	*SCW4*	putative family 17 glucosidase
YGR087C	*PDC6*	indolepyruvate decarboxylase 6
YIR028W	*DAL4*	allantoin permease
YIL166C	*SOA1*	Soa1p
